# Italian healthcare workers' views on mandatory vaccination

**DOI:** 10.1186/1472-6963-9-100

**Published:** 2009-06-11

**Authors:** Silvio ST Tafuri, Domenico DM Martinelli, Giovanni GC Caputi, Annamaria AA Arbore, Cinzia CG Germinario, Rosa RP Prato

**Affiliations:** 1 Department of Biomedical Sciences, Hygiene Section, University of Bari, Apulia Regional Epidemiological Observatory, Bari, Italy; 2 Department of Medical Sciences, Hygiene Section, University of Foggia, Apulia Regional Epidemiological Observatory, Foggia, Italy

## Abstract

**Background:**

Mandatory vaccination has contributed to the success of immunisation programmes but voluntary vaccination allows people to be responsible for their own health. There are benefits from both policies and the arguments between them remain subject to debate within and without the scientific community, both nationally and internationally. The aim of this study is to assess the opinions of those who actually work in the Vaccination Service.

**Methods:**

The survey was carried out using a self-administered standardised anonymous questionnaire given to all of the Vaccination Service employees in the Apulia Region.

**Results:**

Of 302 completed questionnaire replies, 4.4% stated that mandatory vaccination should be abandoned now, 21.2% that it should be phased out, and 74.4% that it should be retained.

**Conclusion:**

An educational program should be set up to explain to Vaccination staff the value and worth of voluntary compared to mandatory vaccination and why high vaccination rates do not have to depend on compulsion.

## Background

Mandatory vaccination was introduced for the first time in the nineteenth century in some European countries following the then sweeping smallpox epidemics [1]. Compulsory vaccination for some diseases is still extant in some countries like France, Greece, Portugal and Belgium; in other countries, like the United Kingdom and Finland, vaccinations are voluntary but the state pursues a policy of active promotion and information-giving, with vaccination being free to the user. In the USA and in Canada vaccinations are not compulsory, but some are a requirement for school enrolment [2].

In Italy, the Jenner anti-smallpox vaccination was made compulsory by law on the 22nd December 1888 [3]. A further health law in 1934 allowed revaccination by the health authorities in the case of a high risk of the spreading of smallpox [4]. With the eradication of the disease, mandatory vaccination was suspended in 1977 and abolished in 1981 [5].

As of today, vaccination, as pre-exposure prophylaxis, is mandatory for infants against diphtheria, tetanus, polio and hepatitis B [6-9].

Currently, within the scientific community, both nationally and internationally, there is an ongoing debate on compulsory vaccination and though it has contributed to the success of immunisation programmes, its benefits are not universally recognised [10]. Rossi L. et al declare that: being duty-bound to vaccination doesn't allow people to develop a "vaccination conscience" and so accept vaccines as the most important effective tool in infectious diseases prevention. But if compulsory vaccination is abolished it's necessary to educate people on health and it's also necessary to share resources and aims in the health world [11].

Recently, the American Academy of Pediatrics concluded that "Continued (vaccine) refusal after adequate discussion should be respected unless the child is put at significant risk of serious harm (as, for example, might be the case during an epidemic). Only then should state agencies be involved to override parental discretion on the basis of medical neglect" [12].

In 2007, because of the specially favourable vaccination program conditions in the Veneto Region of Italy, it's regional government passed a law allowing the experimentation of the abolition of mandatory vaccination within its borders. With a vaccination coverage rate in the last ten years of over 90% for both mandatory and recommended vaccinations, a region-wide computerised register, and a network of vaccination clinics easily reachable by the public, the trial was approved and is being monitored by a panel of experts from both the Italian Ministry of Health and the National Health Institute [13].

However, many of those who work in the field of prevention do not consider it feasible to apply the Veneto experiment to all of Italy or into Europe. Though in some Regions vaccination coverage for the primary immunisation cycle appears sufficiently high to indicate a widespread understanding of the need for vaccination, in other areas the abolition of mandatory vaccination would carry some risk as it could be interpreted as a lack of usefulness of vaccination [14], and not only by the public but also by Vaccination Clinic staff themselves who carry out an irreplaceable role in giving correct, clear and complete information to parents on the benefits and risks of vaccination. In fact, the use of personnel without formal health care training to work with parents on immunization issues is cause for concern because many misconceptions could be transmitted to parents [15]. The recent experience in Italy with the Plan for the Elimination of Measles and Congenital Rubella shows how, with an agreed and well planned program and through the coordination of all the players in the "vaccination" system, it is possible to reach levels of optimal coverage without compulsory vaccination [16]. Currently in Apulia, however, there are no formal discussions between the authorities, the scientific community and the health staff in the Vaccination Centres who have the experience of vaccination and its promotion.

The aim of this study is to evaluate the opinions and perceptions of the staff of the Vaccination Clinics within the Apulia Region to mandatory vaccination and its potential abrogation. The work procedures in operation in the clinics were also investigated, with the aim of identifying those points where intervention would be needed to permit a change to a system based on active promotion and voluntary uptake of vaccination.

## Methods

The study was conducted through a self-administered anonymous questionnaire [see Additional file 1], developed *ad hoc *for this study, standardised and validated through a pilot survey among a limited group of healthcare workers.

The questionnaire reported demographic information, occupation, length of service in the vaccine clinic, the opinion on mandatory vaccination, the procedures for calling the parents to the clinic for the primary appointment and for the issuing of subsequent reminders, the conduct of the health worker when faced with parents who refuse vaccination for their child, and the importance the health worker gave to the computerisation of the vaccination registers.

The questionnaire was given to all the employees of the Apulian Vaccination Services in the period March-May 2008, immediately before each employee began a 10-day training course on the updated management system of the vaccination registers. The employee had 15 minutes to complete the questionnaire.

The data was analysed with the statistical software Epi-Info 6.00.

## Results

With a response rate of 100%, the total number of respondents were 302 health care personnel of the Vaccination Services, 220 (72.8%) women and 82 (27.2%) men, with an average age of 48.9 years (SD = 6.7; range 25–60).

The personnel was composed of doctors (100/302, 33.1%), nurses (175/302, 58%) and health visitors (27/302, 8.9%). The average length of service in the vaccination service was 13.4 years (SD = 9,0), varying from 1 to 43 years of which 23.4% (95% CI = 18.4–28.4) with less than 5 years service, 19.4% (95% CI = 14.8–24.1) from 5 to 10 years, 39.9% (95% CI = 34.2–45.7) with 11–20 years, and 17.3% (95% CI = 12.8–21.7) with more than 20 years service.

Of those questioned, 4.4% (95% CI = 2–6.7) thought that mandatory vaccination should be abolished now, 21.2% (95% CI = 16.6–25.9) that it should be abolished gradually, while the majority of respondents, 74.4% (95% CI = 69.4–79.4), declared that it should be retained. The opinion that mandatory vaccination should be abolished was held by a higher proportion of doctors than by other health staff (Table 1).

**Table 1:** Healthcare workers points of view about mandatory vaccination (%)

	**Medical doctor**	**Other healthcare workers**	**Total**
*mandatory vaccination should be abolished now*	7.1	3.1	4.4

*mandatory vaccination should be abolished gradually*	25.5	18.4	21.2

*mandatory vaccination should be retained*	67.4	78.5	74.4

For primary vaccination appointments, the procedure varied among the clinics, with the most common being the sending of a postcard which informed the parents only of the duty to vaccinate the infant, together with the address and opening times of the clinic. This was in 66.6% (95% CI = 61.2–71.9) of cases, while in 26.8% (95% CI = 21.7–31.8) of cases, the staff preferred to send a letter explaining the importance of vaccination for the infant and for the community, and 4.3% (95% CI = 2–6.7) called the parents to the clinic through the family paediatrician. There were 2.3% (95% CI = 0.6–4.1) of cases in which the staff did not call the parents at all. The use of an explanatory letter of invitation and contact with the paediatrician were more common among doctors (Table 2). Of the respondents, 16.2% (95% CI = 12.1–20.4) declared that they did no follow-through to check if the people who had been called for appointments actually came. This rate was slightly higher among doctors (17.2%; 95% CI = 9.7–24.6) than among other staff (15.6%; 95% CI = 10.5–20.6). In other cases, when the infant was not brought to the clinic, 16.6% (95% CI = 12.4–20.7) stated that they followed up with a single reminder, while 68.2% (95% CI = 62.9–73.5) stated they sent further reminders. These figures can be broken down respectively to 19.2% (95% CI = 11.4–26.9) and 66.7% (95% CI = 57.4–75.9) for doctors, and 14.6% (95% CI = 9.7–19.6) and 74.9% (95% CI = 68.8–80.9) for other staff.

**Table 2:** Most frequently used procedures for primary vaccination (%)

	**Medical doctor**	**Other healthcare workers**	**Total**
postcard	64.6	67.3	66.6

motivational letter	27.3	26.5	26.8

family paediatrician	6.1	3.6	4.3

not call the parents	2	2.6	2.3

When the parents did not bring the child, in 38.7% (95% CI = 33.2–44.2) of cases the family paediatrician was informed, broken down as 42.4% (95% CI = 32.7–52.2) for the doctors and 37.7% (95% CI = 30.9–44.4) for other staff. In other cases (19.2%; 95% CI = 14.8–23.6), as per Italian Law, the City Hall of the town of residence was informed. For doctors this figure was 25.2% (95% CI = 16.7–33.8) while for other staff it was lower at 15.6% (95% CI = 10.5–20.6).

There were 2.3% (95% CI = 0.6–4) of the health staff who believed that the refusal to vaccinate was a parental right and therefore made no effort to compel vaccination. They were 2% (95% CI = -0.7–4.8) of the doctors and 2.5% (95% CI = 0.3–4.7) of the other health staff.

Almost all staff believed it necessary to computerise the vaccination record system, as quickly as possible for 69.8% (95% CI = 64.6–75.1) and gradually for 27.8% (95% CI = 22.7–32.9), while only 2.4% (95% CI = 0.6–4.1) saw no need. In detail these results were respectively, for doctors 66.7% (95% CI = 57.3–75.9), 32.3% (95% CI = 23.1–41.5) and 1% (95% CI = 0.9–2.9), while for other staff they were 71.4% (95% CI = 65.1–77.7), 25.5% (95% CI = 19.4–31.6) and 3.1% (95% CI = 0.6–5.5).

## Discussion and conclusion

The debate between mandatory or voluntary vaccination has been the subject of numerous scientific articles, and there is much information available from anti-vaccination groups, especially on the web [17], but there are few surveys on the opinions of health workers and the general public. A French survey in 2008 of parents paediatricians and general practitioners found that a majority of parents (56.5%) and almost half of doctors (42%) were in favour of mandatory vaccination [18]. This is not discordant with the main finding of our survey that, within Apulia, a large majority of the health staff of the Vaccination Clinics disagree with the abrogation of mandatory vaccination. Clearly our survey is limited by the population under study composed of a group of healthcare staff who work in one sector in one region of Italy. There is no comparison with medical professionals outside the Clinic environment nor with other regions nor with the general public. However, this specific group of health workers has a background in vaccination and their views, though they could be coloured by a conservative attitude towards innovation, are clearly influenced by their experience and can be, we believe, important for the change-over which is taking place in vaccination in the various Health Systems.

The opinion of the Vaccination Clinic staff was clearly reflected in the mode of operation for the primary appointment. Most of staff in fact use a simple postcard to call the infants to the clinic. This postcard uses sentences such as "*as required by law you are invited to come to the Vaccination Clinic of this town to carry out the mandatory vaccinations*", together with the address and opening hours. Often included are comments such as "*Non-compliance will be reported to the authorities*."

While almost 70% declared that they issued multiple reminders, over 30% declared that there was only one reminder issued or no follow up at all, which is certainly not good practice, though this was slightly compensated for by the number of staff, 38.7%, who declared that they contacted the family paediatrician in the case of the infant not coming to the clinic and this is the gold-standard in health care. Unfortunately, the respondents following this policy were in a minority, even though the paediatrician has an irreplaceable role in advising the parents on health care choices, especially when the parents don't bring the child to the clinic or they refuse to vaccinate, either because of lack of awareness of the importance of vaccination, or, as often happens, mistaken beliefs about it. The synergy between Public Health and the health care workers in direct patient contact should be part of the fundamental strategy for the activities of the Vaccination Service [19].

The secondary finding from the survey is that the staff of the Vaccination Clinics still live in a bureaucratic world as can be seen from the primary appointment policy, from the lack of synergy with the paediatrician and from the use of the out-dated and inefficacious application of a fine to the parents.

In Italy, there are several old laws, especially from the post-war period, enacted for disease prevention or health protection, which are now outdated, either because Evidence Based Prevention has disproved previous beliefs or because the original risk no longer exists. These norms and their practice in the Health Service create ritual actions that the citizen sees only as useless bureaucratic machinations, so creating mistrust and causing a waste of resources both for the Health Service and for the community.

The abolishing of mandatory vaccination would create a dilemma for the Health Service seeming to belie that a compulsory procedure as vaccination is of the highest importance, while public perception, and even that of Health Service staff, could be that vaccination has served its usefulness and is now out-dated, such as has happened to the "Certificate of Good Health" previously necessary for school admission.

Offering vaccination to the public should be one of the jewels in the crown in the service of health promotion for infants, displacing the idea that it is a bureaucratic necessity. A good start would be to eliminate, first in health care staff and then in the public mind, the categorisation of vaccinations as either mandatory or recommended, a distinction which only creates hierarchies of priority which are not functional in achieving the objectives of public health care.

The picture which emerges from this study is one of staff blinkeredness to the idea of abolishment of compulsory vaccination, an idea which they probably view as underestimating the value of their work because it could cause the disappearance of the health promotion activities traditionally brought to the public through vaccination. This fear can be justified in part by their length of service and by their age which, on average, is nearly fifty.

The abolishment of mandatory vaccination, which requires a series of prerequisites both cultural and organisational, would be a challenge for a country like Italy which would risk a collapse of vaccination coverage in the weaker areas or even the loss of control of such a complex and delicate activity. To move to the future, it is necessary to give suitable educational training to vaccination staff that can make clear to them the intrinsic worth of changing from mandatory to informed vaccination. This is a responsibility for the Health Service which can bring it closer to its public and to parents who instead of being passive users can become responsible for their own health care and that of their children and so not let them fall prey to preventable disease.

## Competing interests

The authors declare that they have no competing interests.

## Authors' contributions

ST and RP conceived the study, participated in its design, performed analysis and interpretation of data and drafted the manuscript. DM contributed to analysis and interpretation of data. GC and AA contributed to conception of the study. CG conceived the study and contributed to interpretation of data. All authors read and approved the final manuscript.

## Pre-publication history

The pre-publication history for this paper can be accessed here:



## Supplementary Material

Additional file 1 **Questionnaire**. The file provided represent the questionnaire used in the survey. Click here for file
