# Supplementary material for: Italian healthcare workers' views on mandatory vaccination
Source: BMC Health Serv Res. 2009 Jun 11;9:100. doi: 10.1186/1472-6963-9-100 (PMC2706237; doi:10.1186/1472-6963-9-100)
Supplement: Additional file 1 — Questionnaire. The file provided represent the questionnaire used in the survey. [file 1472-6963-9-100-S1.doc]

**QUESTIONNAIRE**

**Personal and professional information**

1 Sex ⬜ Female ⬜ Male

2. Age _______

3 Length of service in the Vaccine Service ________

4 Hospital Trust ____________

5 Profession ⬜ Doctor ⬜ Nurse ⬜ Health visitor

6 Year when you qualified ___________

*(give the year of your first professional qualification)*

**Your opinion regarding vaccination**

7 You believe mandatory vaccination ⬜ is necessary/ should be retained

⬜ should be abandoned

⬜ should be phased out

8 Your opinion on the vaccinations which are included in the regional program but not in the national one

Pneumococcus

Not at all important 1 ⬜ 2 ⬜ 3 ⬜ 4 ⬜ 5 ⬜ 6 ⬜ 7⬜ Very important

Meningococcus C

Not at all important 1 ⬜ 2 ⬜ 3 ⬜ 4 ⬜ 5 ⬜ 6 ⬜ 7⬜ Very important

Varicella

Not at all important 1 ⬜ 2 ⬜ 3 ⬜ 4 ⬜ 5 ⬜ 6 ⬜ 7⬜ Very important

Hepatitis A

Not at all important 1 ⬜ 2 ⬜ 3 ⬜ 4 ⬜ 5 ⬜ 6 ⬜ 7⬜ Very important

9 The right of each Region to add vaccinations to the national vaccination program

⬜ is necessary/ should be retained

⬜ should be abandoned

⬜ should be phased out

1. Your opinion regarding the administration of more than one vaccine at a time:

helps the organisation of the vaccine centre ⬜ yes ⬜ no

increases vaccination take-up ⬜ yes ⬜ no

increases the risk of adverse reactions ⬜ yes ⬜ no

frightens the parents ⬜ yes ⬜ no

1. In your centre do you practise co-administration of vaccines?

⬜ Yes ⬜ No

*If you answered No, go to question 13*

1. Which co-administration do you carry out?

⬜ Hexavalent + Pneumococcus heptavalent

⬜ MenC + Varicella

⬜ Varicella + Hepatitis A

⬜ MenC + Hepatitis A

⬜ Other (specify) _________

13 Your opinion regarding the computerisation of the vaccine registers

⬜ it is necessary to complete the work immediately

⬜ it is necessary to complete the work gradually

⬜ it increases the work-load of the vaccine centres

⬜ it would be better to postpone the work to allow a better organisation of the service

14 How do you carry out calling for a vaccination appointment?

⬜ post-card

⬜ letter with an explanation of the need for vaccination

⬜ in agreement with the family paediatrician

⬜ in my centre there is no calling for appointment

15 If a child does not come to the appointment

The work organisation does not allow me to follow up ⬜ yes ⬜ no

I send a single reminder ⬜ yes ⬜ no

I send multiple reminders ⬜ yes ⬜ no

I contact the family paediatrician ⬜ yes ⬜ no

I inform the City Hall of the home town ⬜ yes ⬜ no

I do nothing, it is the parents’ choice ⬜ yes ⬜ no

16 Your opinion regarding the HPV vaccination

Not at all important 1 ⬜ 2 ⬜ 3 ⬜ 4 ⬜ 5 ⬜ 6 ⬜ 7⬜ Very important

**Vaccination of health workers**

17 Influenza vaccination for health workers

Not at all important 1 ⬜ 2 ⬜ 3 ⬜ 4 ⬜ 5 ⬜ 6 ⬜ 7⬜ Very important

18 Hepatitis B vaccination for health workers

Not at all important 1 ⬜ 2 ⬜ 3 ⬜ 4 ⬜ 5 ⬜ 6 ⬜ 7⬜ Very important

19 Varicella vaccination for susceptible health workers

Not at all important 1 ⬜ 2 ⬜ 3 ⬜ 4 ⬜ 5 ⬜ 6 ⬜ 7⬜ Very important

20 MMR – measles, mumps, rubella vaccination for susceptible health workers

Not at all important 1 ⬜ 2 ⬜ 3 ⬜ 4 ⬜ 5 ⬜ 6 ⬜ 7⬜ Very important

21 BCG vaccination for susceptible health workers

Not at all important 1 ⬜ 2 ⬜ 3 ⬜ 4 ⬜ 5 ⬜ 6 ⬜ 7⬜ Very important

22 In the 2007/2008 season did you receive at least one influenza vaccination?

⬜ YES ⬜ NO

23 Who **proposed**/administered the influenza vaccination?

⬜ Industrial medical service

⬜ Occupational health physician

⬜ No-one/I decided myself

24 Have you received the Hepatitis B vaccination?

⬜ YES ⬜ NO

25 Who **proposed**/administered the Hepatitis B vaccination?

⬜ Industrial medical service

⬜ Occupational health physician

⬜ No-one/I decided myself

26 Have you received the Varicella vaccination?

⬜ SI ⬜ NO

If no

Are you susceptible to varicella ⬜ YES ⬜ NO

27 Who **proposed**/administered the Varicella vaccination?

⬜ Industrial medical service

⬜ Occupational health physician

⬜ No-one/I decided myself

28 Have you received the MMR vaccination?

⬜ SI ⬜ NO

If no

Are you susceptible to one of the three diseases ⬜ YES ⬜ NO

29 Who **proposed**/administered the MMR vaccination?

⬜ Industrial medical service

⬜ Occupational health physician

⬜ No-one/I decided myself

30 Have you received the BCG vaccination? ⬜ YES ⬜ NO

If no

Mantoux positive ⬜ YES ⬜ NO

29 Who **proposed**/administered the Mantoux and potential vaccination?

⬜ Industrial medical service

⬜ Occupational health physician

⬜ No-one/I decided myself
